# A pathway to strengthening open science: comments on the draft *South African Ethics in Health Research Guidelines*

**DOI:** 10.3389/fphar.2024.1304950

**Published:** 2024-03-01

**Authors:** Amy Gooden

**Affiliations:** School of Law, University of KwaZulu-Natal, Durban, South Africa

**Keywords:** data, genomics, health research, open access, open science, policy, South Africa

## Abstract

The recently released *draft South African Ethics in Health Research Guidelines: Principles, Processes and Structures* (Draft Guidelines) by the National Health Research Ethics Council recognize open data and provide guiding principles for this in the context of health research in South Africa. While its inclusion is a positive development, there is room for improvement. Although the Draft Guidelines leverage the *Draft National Policy on Data and Cloud*, it lacks incorporation of other relevant government policies, notably the *Draft National Open Science Policy*, and fails to sufficiently detail the principles of open science and open access. This limited scope and lack of comprehensive definition and detailed guidance present challenges for researchers in conducting ethical and responsible health research in South Africa. It constrains the Draft Guidelines from fully aligning with national imperatives and from fostering African-centric approaches. To address these issues, it is recommended that the Draft Guidelines integrate broader policies and principles, enhance clarity through comprehensive definitions, provide detailed guidance on open access, and promote African-centric approaches. Implementing these solutions will strengthen the Draft Guidelines, aligning them with national visions of open science, and thereby harnessing the full potential of South Africa’s diverse scientific community in advancing health research.

## 1 Introduction

In recent years, there has been a proliferation of health research worldwide. Health research contributes to the understanding of disease, the improvement of healthcare systems, the development of new medicines and treatments, and technologies aimed at bettering health and healthcare (DoH, 2015). As such, health research has the potential to benefit the population—especially in South Africa where there is a high disease burden, predominantly from HIV/AIDS and tuberculosis (Abdool Karim et al., 2009; Mayosi et al., 2012; Johnson et al., 2017; Abdool Karim and Baxter, 2022; Kubjane et al., 2022).

With the growth of health research in South Africa came the need to address various ethical concerns in health research, align with international standards, protect research participants, and ensure the proper conduct of health research. In 2015, the Department of Health (DoH) released the second edition of the *Ethics in Health Research: Principles, Processes and Structures* (DoH, 2015) (DoH Ethics Guidelines), to replace the previous 2004 edition. The DoH Ethics Guidelines provide guidance to health researchers in South Africa and cover certain key aspects of health research, such as informed consent, the need for ethical review, community engagement, benefit sharing, risk assessment, the protection of research participants’ rights, and the upholding of ethical principles like autonomy and privacy (DoH, 2015). Importantly, the DoH Ethics Guidelines are not simply “soft law”; they are made legally binding by regulation 2(a) of the Regulations Relating to Research with Human Participants (GN R179 of GG 38000, 2014)—therefore, health researchers in South Africa are legally compelled to comply with the DoH Ethics Guidelines.

Health research has further progressed with the advancement of genome sequencing, which led to genomics research and the use of large datasets. The availability of health research data, which could have huge positive impacts on population health, led to calls for datasets, materials, processes, protocols, findings, results, and software to be made more accessible (Spellman et al., 2017; Ramachandran et al., 2021; Chakravorty et al., 2022). Although the idea of open science has existed for many years and was adopted when science research in universities was thriving (Baca, 2006; Rhoten and Powell, 2007; Scaria and Rangarajan, 2016; Krishna, 2020), in recent years open science has come under pressure due to intellectual property law and policy developments, which has caused research to become commercial and proprietary instead of open (Baca, 2006). However, health research (inclusive of genomics research) has driven calls for the promotion of open science given the vast amounts of data generated by genomics research and the need for collaborative efforts in order to analyze it (International Human Genome Sequencing Consortium, 2001), the need for reproducibility and transparency (Begley and Ellis, 2012), the promise of precision medicine (Collins and Varmus, 2015), and the potential for increased discoveries to be made with access to more data (Venter et al., 2001). The data-intensive, collaborative, and translational nature of health and genomics research has led to it being a driving force in advocating for open science (Hetu et al., 2019; Staunton et al., 2021). Not only does open science accelerate research, but it also lessens the wastage of research resources (Buxton et al., 2021), allows the inspection of research outputs (Besançon et al., 2021), enhances transparency, research integrity, and the responsible use of genomic data (Grant et al., 2022; Haven et al., 2022).

The newly released *draft South African Ethics in Health Research Guidelines: Principles, Processes and Structures* by the National Health Research Ethics Council (NHREC) (NHREC, 2023) (Draft Guidelines), which were circulated amongst stakeholders for comment, are an attempt to revise the second edition 2015 DoH Ethics Guidelines and develop a third edition—giving South Africa with an opportunity to provide guidance for open science in health research, something which was not addressed in the 2015 DoH Ethics Guidelines. This article provides a commentary on the Draft Guidelines, focusing on its handling of open science and open access data. In this article, I highlight several problematic aspects of the Draft Guidelines and suggests potential solutions. I begin by introducing open science broadly, and then examining the concept in South Africa specifically. Thereafter, I analyze the Draft Guidelines’ addition of guiding principles for open access, identifying where the Draft Guidelines have succeeded in providing clear guidance, as well as areas in which the Draft Guidelines are lacking. Throughout this article, I provide a way forward for the promotion of open science in South Africa, and emphasize areas where the Draft Guidelines can improve in this regard. Given that there have been recent academic pushes for openness, specifically in genomics research in South Africa (Gooden and Thaldar, 2023a; Thaldar et al., 2023a; Gooden and Thaldar, 2023b), it is imperative that this issue be given due consideration.

## 2 The imperative for open science

Given that advancements in technology have allowed science to become more “open,” open science must be viewed as distinct from the previous *status quo* where, for example, publications were only available to subscribers of journals post publication (Friesike et al., 2015). Various definitions have been utilized to describe open science and what it entails. Broadly, open science aims to make research methods and results freely available in order to promote collaboration and transparency to the benefit of the community (Strydom et al., 2022). However, open science should be distinguished from open access. Open access—as a practice of open science—is a set of principles and procedures allowing research outputs to be freely accessible, without any costs or other access barriers (DSI, 2022). Open access allows for published work to be obtained, while open science provides access to the whole scientific knowledge process (Heise and Pearce, 2020).

A common definition of open science, put forward by Maurer (2003), is that it “tends to connote (a) full, frank, and timely publication of results, (b) absence of intellectual property restrictions, and (c) radically increased pre- and post-publication transparency of data, activities, and deliberations within research groups”. Vicente-Saez and Martinez-Fuentes (2018) define open science as “transparent and accessible knowledge that is shared and developed through collaborative networks”. Open science is seen to comprise of certain central elements, such as (a) open data, (b) open source, (c) open access, (d) open material, (e) open peer-review, and (f) open educational resources (Levy et al., 2010; Krishna, 2020). In many definitions of open science, there are certain common terms that often feature. These include: (a) open, (b) transparent, (c), accessible, (d) shared, (e) collaborative, (f) available, and (g) replicable (Scaria and Rangarajan, 2016; Vicente-Saez and Martinez-Fuentes, 2018). Open science is vital in advancing research, innovation, and society. It emphasizes accessibility, collaboration, and transparency (Nielsen, 2011; Gewin, 2016). Through open science, the sharing of data, methods, and findings makes research more accessible and reproducible, which enhances scientific discovery, democratizes access to knowledge, grows research impact, and increases public trust in science (Fecher and Friesike, 2014; Nosek et al., 2015; McKiernan et al., 2016; Hardwicke et al., 2018).

Central to the implementation of open science is the FAIR Guiding Principles, which are applicable to scientific data management and stewardship (Wilkinson et al., 2016). These principles aim to minimize barriers to research outputs, thereby allowing others to discover, understand, and re-use such outputs—which may lead to further findings and opportunities, as well as take advantage of existing resources (UCL, 2024). FAIR stands for: (1) findability, which aims to make research more easily discoverable; (2) accessibility, which entails information on how to access the data; (3) interoperability, which allows the data to be integrated with other data; and (4) reusability, which allows for research outputs to be repurposed (Wilkinson et al., 2016; UCL, 2024). In addition to the FAIR Guiding Principles are the CARE Principles for Indigenous Data Governance. The CARE Principles are people centered, and aim to ensure that research is done in such a way so as to benefit indigenous people, and to highlight the how data can further the innovation and self-determination of indigenous people (GIDA, 2019; DSI, 2022). CARE stands for: (1) collective benefit, where data ecosystems should allow indigenous people to derive benefit from the data (GIDA, 2019); (2) authority to control, which recognizes and allows indigenous people to control their data (GIDA, 2019); (3) responsibility, which requires those working with indigenous data to publicize the ways in which the data is used to promote indigenous people’s self-determination and collective benefit (GIDA, 2019); and (4) ethics, which ensures that the rights and wellbeing of indigenous people is central in all research endeavors (GIDA, 2019). In South Africa, open science has been defined as “research and development that is collaborative, transparent and reproducible and whose outputs are publicly available” (DSI, 2022). The Department of Science and Innovation (DSI), previously the Department of Science and Technology (DST), in its *White Paper on Science, Technology and Innovation* (STI White Paper), provides that open science “refers to an approach to research based on greater access to public research data enabled by information and communications technology (ICT) tools and platforms, broader collaboration in science–including the participation of non-scientists–and the use of alternative copyright tools for diffusing research results” (DST, 2019). The African Open Science Platform (AOSP) recognizes that open science tends to refer to open data and open access publishing (AOSP, 2023). However, the AOSP notes that this only provides a limited view of what open science actually is. Open science is not limited to scientists, but should be a more public enterprise that includes the public and private sector, business, policymakers, government, communities, and citizens who engage with scientists to explore solutions to issues facing society (AOSP, 2023).

Open science has not only been promoted by the AOSP in various strategies and reports (ASSAf, 2019; AOSP, 2023), but it is also the subject of the *Draft National Open Science Policy*, which was shared by the DSI with stakeholders in 2022. The Draft National Open Science Policy aims to democratize scientific knowledge and thereby strengthen the research landscape by making research outputs accessible, advancing economic development, and promoting research collaboration (DSI, 2022). The Draft National Open Science Policy is guided by various principles, such as findability, accessibility, reusability, transparency, responsibility, flexibility, and sustainability (DSI, 2022). Open science also features in the STI White Paper, where ideas such as inclusivity, innovation culture, and policy coherence are introduced in order to promote science, technology, and innovation while addressing global challenges like the Fourth Industrial Revolution (DST, 2019). Open science is recognized as a means through which the benefits of collaborative, transdisciplinary approaches to knowledge development, as well as the spread of ideas and research, may be realized (DST, 2019).

Given the importance of open science, one would expect it to appear in most government documents. However, in South Africa, a focus on open science has been lacking, and it has not featured in many recent and relevant publications—such as the *Draft National Policy on Data and Cloud* (Department of Communications and Digital Technologies, 2021), the Protection of Personal Information Act 4 of 2013, 2013 (POPIA) Code of Conduct for Research (ASSAf, 2023), and the *Bio-Economy Strategy* (DST, 2013), to name a few. The Draft Guidelines are no exception—any mention of open science and its promotion in health research in South Africa is absent from the Draft Guidelines. This, I suggest, is a missed opportunity and one that should be addressed by the NHREC.

## 3 Analysis of the Draft Guidelines

The Draft Guidelines are intended to provide minimum standards for undertaking ethical and responsible research in South Africa (NHREC, 2023). They cover different types of health research, guiding principles for ethical research, processes for ethics review, research ethics committees, health research ethics infrastructure, as well as human biological material and data used in research (NHREC, 2023). Unlike the 2015 DoH Ethics Guidelines, the Draft Guidelines provide principles for open access in health research (NHREC, 2023). This is important because it ensures that valuable knowledge—which may be crucial in bettering population health and developing cures and treatments for disease—is freely available (Smith et al., 2017; Day et al., 2020; Strydom et al., 2022). The inclusion of open access in the Draft Guidelines initially appears as a promising step forward considering South Africa’s commitment to open science, which has featured in the STI White Paper, and formed a central part of the Draft National Open Science Policy and the STI White Paper. However, despite having the opportunity to further promote open science and open access databases in South Africa, the Draft Guidelines only refer to the Draft National Policy on Data and Cloud—a policy that, although positive in its vision to facilitate free access to data, has been criticized for the means to achieve it, which entails government control of access to data, nationalizing all data generated in South Africa, and interrupting the intellectual property legal framework (Thaldar et al., 2023b). As such, the Draft Guidelines fail to provide a comprehensive and inclusive pathway for open access databases, and thereby open science, in research in South Africa.

In what follows, I analyze various problematic aspects of the Draft Guidelines, specifically in relation to open science—namely, the failure to consider open science, the definition of open data, the importance of comprehensive definitions, the matter of privacy and consent, and the failure to provide proper guiding principles for open access data—and point towards potential solutions, where relevant.

### 3.1 The failure to consider open science

Open data, which is explicitly referred to in the Draft Guidelines, is regarded as a “sub-set” of open science (ASSAf, 2019). Concepts such as open data, open access, and open source are all considered within the practice of open science (Strydom et al., 2022). Therefore, open data—which is mentioned in the context of research—should not be discussed without considering the broader framework of open science. This is something that has been recognized and promoted by the Draft National Open Science Policy, but which the NHREC appears to overlook. However, the Draft Guidelines fail to address open science, and thereby negate a vital aspect of research in South Africa.

In recent years, there has been a push for open science in South Africa and the concept has featured in two government documents: The 2019 STI White Paper and the 2022 Draft National Open Science Policy. However, the Draft Guidelines only focus on one aspect of open science—namely open data—and fail to even mention open science. Therefore, the Draft Guidelines do not promote government policies and strategies intended to further research in South Africa and make it more open and accessible.

However, it should be noted that, without expressly stating so, the Draft Guidelines do appear to point towards open science. The Draft Guidelines recognize that the sharing of data has the potential to *inter alia* enable broad dissemination of research results, increase collaboration, enhance responsiveness to challenges in society, encourage research integrity, and promote greater transparency (NHREC, 2023). In essence, this is open science. Yet, principles that are aligned with open science—such as reproducibility, transparency, and translatability—seem to only apply in the context of animal research and not in terms of research with human participants (NHREC, 2023). Further, international collaboration and the sharing of funding, knowledge, and data—all vital to open science—are only mentioned in the context of public health emergencies, such as the COVID-19 pandemic and not as the norm (NHREC, 2023). It appears as if the Draft Guidelines implicitly recognize open science and its importance, but only in certain contexts such as genomics research, research on animals, and public health emergencies. I suggest that it would be beneficial for the Draft Guidelines to consider explicitly mentioning open science and expanding on its importance in health research, especially given the existence of government policies and strategies that promote it.

### 3.2 Defining a sub-set of open science: open data

Generally, definitions of open data denote that such data must be freely accessible to be used and re-used by anyone (Scott, 2017; European Commission, 2023; Open Data Charter, 2023; Open Data Handbook, 2023; Open Knowledge Foundation, 2023)—with the only restriction being acknowledgement of the source or share-alike (Open Data Handbook, 2023; Open Knowledge Foundation, 2023).

The Draft Guidelines rely on the definition of “open data” provided in the Draft National Policy on Data and Cloud, which it defines as “data that is made freely available to everyone for use, re-use and republishing as they wish, subject to ensuring protection of privacy, confidentiality and security in line with the Constitution” (Department of Communications and Digital Technologies, 2021). Yet, this is not the only definition of open data available. Although similar, the Draft Guidelines exclude the definition of “open data” provided in the Draft National Open Science Policy, which it defines as “data that anyone can freely access, use and share, subject, at most, to requirements that preserve provenance and openness” (DSI, 2022). Additionally, the *National Integrated ICT Policy White Paper* (ICT Policy White Paper) defines open data as “datasets that can be freely used, re-used and distributed by anyone, only subject to (at the most) the requirement that users attribute the data and that they make their work available to be shared as well” (Department of Telecommunications and Postal Services, 2016). Having regard to these other definitions of open data that exist would provide researchers with a more comprehensive idea of how open data has been defined by various South African government departments. Therefore, I suggest that the Draft Guidelines develop their own definition of “open data”—that aligns with its objectives—but that references those found in the Draft National Policy on Data and Cloud, the Draft National Open Science Policy, and the ICT Policy White Paper.

### 3.3 The importance of comprehensive definitions

The provision of definitions serves to assist in providing a common understanding of key terms, thereby lessening the chance of ambiguity and misinterpretation, and ensuring consistent implementation (Whitfield, 2012; Podsakoff et al., 2016). In terms of policies and guidelines, a lack of clear and comprehensive definitions leads to a lack of clarity, which may impede the achievement of policy objectives.

The Draft Guidelines lack definitions relevant to open access data, and only contain a definition of “open data” (defined above). However, had the Draft Guidelines placed this within the broader concept of open science, a definition of such would have been beneficial. Notwithstanding this, there are other definitions relevant to open access and data in research that are pertinent to include. For example, the Draft National Open Science Policy defines “open access” as “a set of principles and a range of practices through which research outputs are distributed online, free of cost or other access barriers” (DSI, 2022). This is highly relevant to research in general, and health research specifically. In considering openness, it is not only the data that is relevant, but also the accessibility of such data. Therefore, I suggest that the provision of additional definitions—such as “open access”—in the Draft Guidelines would assist in this regard.

Additionally, the Draft Guidelines seem to make fundamental errors in basic definitions. The terms “open data” and “open access” are not synonymous and should therefore be distinguished. However, the Draft Guidelines refer to “open access,” “open data,” and “open access data” and appear to conflate these three terms—which causes confusion regarding what is being referred to (NHREC, 2023). “Open data” refers to the data itself that is made freely accessible, while “open access” denotes principles and practices that allow the free sharing of research outputs (which may be inclusive of data). However, the Draft Guidelines only provide a definition of “open data”—which was adopted from the Draft National Policy on Data and Cloud. I suggest that if the Draft Guidelines had regard to other highly relevant policies that deal with open science, open data, and open access—such as the Draft National Open Science Policy—it would be clear that further definitions exist, and which could have been utilized in the Draft Guidelines in order to clarify the different terminology used.

A further point to note is the differences between the two definitions of “open data”—one provided in the Draft National Open Science Policy and the other in the Draft National Policy on Data and Cloud (and utilized in the Draft Guidelines). Both definitions refer to data that is freely available to all and can be used and shared—although the Draft National Policy on Data and Cloud refers to re-use and republishing (Department of Communications and Digital Technologies, 2021), while the Draft National Open Science Policy uses the term “share” (DSI, 2022). However, the second part of both definitions contain a caveat—in the Draft National Policy on Data and Cloud that the rights to privacy, confidentiality, and security as enshrined in the Constitution are protected (Department of Communications and Digital Technologies, 2021), and in the Draft National Open Science Policy that provenance and openness are preserved (DSI, 2022). These parts of the different definitions appear to be at odds: One promotes openness with very little restriction, and the other allows openness, but only insofar as it does not violate rights to privacy, confidentiality, and security. Although the flaws inherent in the definition of “open data” stem from the Draft National Policy on Data and Cloud, its inclusion in the Draft Guidelines means that this antithesis extends to the health research context—where the privacy rights of research participants have come into question given the nature of genomics research where privacy cannot always be guaranteed (Lunshof et al., 2008; Prainsack and Buyx, 2013; Wang et al., 2017).

Given the above, I suggest that the Draft Guidelines consider revising the definitions provided in relation to open access data. The inclusion of additional relevant definitions—such as open science and open access—as well as the provision of a comprehensive and integrated definition of open data will serve to provide greater clarity when interpreting the Draft Guidelines.

### 3.4 The matter of privacy and consent

Central to health research is the sharing of data and results. Increased access to such data serves to streamline the research process, making it more efficient and participatory by lessening duplication as well as the costs associated with the creation, transfer, and re-use of data (NHREC, 2023). However, on the face of it, such openness seems to be in opposition to privacy. The Draft Guidelines state that there is a “trade-off between protecting privacy and advancing research” (NHREC, 2023). I suggest that positing the interaction between protecting privacy and advancing research as a “trade-off” is a mischaracterization. It is a common myth in the South African context that research is somehow stymied by the new data privacy legislation, POPIA. Respecting privacy rights and advancing research are perfectly compatible, and ought not be conceived of as necessarily in opposition (Thaldar and Townsend, 2020).

The Draft Guidelines also note that although many participants may not want to publicize their health and genetic data, there are some that do and there should be no obstacles to prevent participants, who wish to share their data in an identifiable manner, from doing so—provided that all foreseeable harms resulting from identification are negligible and understood by participants (NHREC, 2023). What is important is that there be an understanding and those that choose to share their data openly do so knowing that their privacy can no longer be guaranteed.

Given the complexities of health and genomics research, as well as the potential risks involved, consent is vital in all health research involving human participants. The Draft Guidelines provide for three types of consent—specific (or narrow) consent, tiered (or differentiated) consent, and broad consent (NHREC, 2023). The Draft Guidelines also mention blanket consent but, where the 2015 DoH Ethics Guidelines stated that blanket consent was “not recommended” (DoH, 2015), the Draft Guidelines do not permit blanket consent as it “cannot sustain fundamental ethical principles, especially that of protection of privacy” (NHREC, 2023). While these modes of consent are relevant, an additional mode of consent that is aligned with the idea of open science is open consent. Open consent was developed by the Harvard Personal Genome Project (PGP) in response to the recognition that, given the nature of genomics research, privacy cannot be guaranteed (Lunshof et al., 2008). It therefore entails individuals openly donating and sharing their data for research without any assurances of anonymity, privacy, or confidentiality (Lunshof et al., 2008). To ensure that consent is informed, individuals are made aware of the benefits and risks of participation (Lunshof et al., 2010), and are additionally required to pass (with full marks) an assessment that tests their understanding of genomics and privacy (Angrist, 2009). By doing away with any expectations of privacy and taking extra steps to ensure that consent is informed, open consent may offer a potential solution to the contention between open access and privacy. Open consent can essentially be viewed as a type of blanket consent to making data open access, as well as an assessment ensuring that the consent is informed (Gooden and Thaldar, 2023a). However, open consent does differ from blanket consent in certain respects. First, while blanket consent may be utilized for data that has been de-identified, open consent makes no such guarantees, and the publishing and sharing of data is unrestricted and identifiable. Second, open consent can be seen to go a step further than blanket consent in requiring potential participants to pass an assessment in order to ensure that consent is informed. Therefore, open consent furthers open science by combining it (and its benefits) with informed consent.

A potential legal and ethical pathway for an open consent model for genomics research and open access databases in South Africa has already been established (Gooden and Thaldar, 2023a; Thaldar et al., 2023a; Gooden and Thaldar, 2023b). Using this as guidance, I suggest that the Draft Guidelines consider the inclusion of such a model as a means to further open science. Furthermore, I suggest that the Draft Guidelines retain the previous provision regarding blanket consent from the 2015 DoH Ethics Guidelines, where blanket consent was not recommended, but was also not prohibited (DoH, 2015). This provides for the possibility of allowing open consent in health research in South Africa.

### 3.5 Failure to provide proper guiding principles for open access data

The Draft Guidelines deal with, what it refers to as, “guiding principles for open access”. The Draft Guidelines provide that because the Draft National Policy on Data and Cloud supports open access to data, there is a need for guiding principles for health research. Contrary to what is stated in the Draft Guidelines, it is not only the Draft National Policy on Data and Cloud that supports open access to data. Other policies and reports—such as the Draft National Open Science Policy (DSI, 2022), the POPIA Code of Conduct for Research (ASSAf, 2023), the Academy of Science of South Africa (ASSAf) report on *Human Genetics and Genomics in South Africa: Ethical, Legal and Social Implications* (ASSAf, 2018) (ASSAf Report), the STI White Paper (DST, 2019), the *Synthesis Report: South Africa Foresight Exercise for Science, Technology and Innovation* (DSI, 2019) (Synthesis Report), the *Bio-Economy Strategy* (DST, 2013), and the ICT Policy White Paper (Department of Telecommunications and Postal Services, 2016)—also promote *inter alia* open access and open data and some provide pathways for doing so. It is true that there may be a need for principles governing open access data for health research, but it must be questioned why the Draft Guidelines have only used the Draft National Policy on Data and Cloud as its basis for doing so.

Before examining each of the guiding principles for open access in the Draft Guidelines, it should be noted that some of the principles in the Draft Guidelines come from the *Concordat on Open Research Data* (Rylance et al., 2016). This concordat was developed by stakeholders in the United Kingdon (UK) and designed for the UK research community. As such, some of the principles for open access adopted in the Draft Guidelines may not align with South Africa’s research space and the principles of open science that are promoted in the country.

#### 3.5.1 Principle (1): data curation is required to preserve data with acknowledged long-term value

Data curation is important in promoting open access (and thereby open science) in research as it maintains the integrity and value of open data. However, the concept of curation is broad and multifaceted, ranging from the selection of data to its management (Lee and Stvilia, 2017). The Draft Guidelines use the term “data curation” in relation to open access, but fail to define it. Further, the Draft Guidelines, in a separate section, require the Principal Investigator to comply with POPIA in terms of *inter alia* data curation (NHREC, 2023). However, there is no mention of data curation in POPIA or in the POPIA Code of Conduct for Research. On the other hand, the Draft National Open Science Policy does refer to data curation. Although not defined, the Draft National Open Science Policy recognizes that those responsible for funding research must also ensure funding for *inter alia* data curation (DSI, 2022). The Draft National Open Science Policy also notes that open science infrastructure is vital in long-term data curation (DSI, 2022). Given the range of meanings that data curation may have, I suggest that it would be beneficial for the Draft Guidelines to provide a definition of their interpretation of “curation” in order to provide clarity to researchers.

The Draft Guidelines mention the preservation of data with “acknowledged long-term value” (NHREC, 2023). But how will this long-term value be determined? Given the nature of health and genomics research that requires vast amounts of data, which can be used and then re-used for different projects, does all data not have some sort of long-term value? Additionally, it cannot be said that data, which is viewed as having little value now, will not be hugely invaluable at some point in the future—especially given the rate at which technology is advancing, and sometimes in unpredictable ways. As such, it does not seem practical or feasible to determine the long-term value of data used in research. Similar to the Draft Guidelines, the Draft National Open Science Policy makes mention of long-term. However, it refers to “long-term data curation” (DSI, 2022), rather than the curation of data with long-term value (NHREC, 2023). The Draft National Open Science Policy also provides a means of ensuring long-term data curation, namely, through data management plans (DSI, 2022).

Although data management plans tend to focus on active research, and long-term data curation deals with the preservation, maintenance, and accessibility of data after the research has been completed (Lee and Stvilia, 2017; NIH, 2023), it is often beneficial to include long-term data curation within a data management plan. This ensures proper planning, visibility and accountability, adequate resource allocation, and provides a consolidated guide that encompasses both current and long-term data management (Coresignal, 2021; UCLA, 2023). Depending on the nature of the research, the type of data collected and its intended use, the research objectives, data sharing, the complexity of the data, and ethical and legal considerations, data curation may need to be more detailed, and may even require a separate document (Lee and Stvilia, 2017; Miller, 2023).

To provide greater clarity to researchers, I suggest that the Draft Guidelines amend this principle to be more in line with the Draft National Open Science Policy. There are two possible ways in which this can be achieved: (1) the Draft Guidelines amend the current principle to “strategies for long-term data curation are required”; or (2) the Draft Guidelines remove the current principle and combine it with principle (4) regarding data management plans, which is discussed below. I suggest that each of the guiding principles for managing open access data provided in the Draft Guidelines contain an explanation in order to expand on the principle and provide proper, and more detailed, guidance to researchers. Therefore, in terms of (1), the Draft Guidelines can explain that detailed long-term data curation may not be required for all research projects, and it depends on the research. In terms of (2), the Draft Guidelines can specify that long-term data curation be included as part of the data management plan—in line with the Draft National Open Science Policy—or, where required and depending on certain factors like the nature of the research and the type of data collected, long-term data curation be detailed separately.

#### 3.5.2 Principle (2): the right of creators of research data to reasonable first use should be recognized

The principle relating to reasonable first use in the Draft Guidelines was adopted from the UK Concordat on Open Research Data (Rylance et al., 2016). Unlike the UK Concordat on Open Research Data, the Draft Guidelines provide no explanation as to what this principle entails. It is evident that a move towards open science requires the sharing of many aspects of research, including original data. According to the UK Concordat on Open Research Data, this may deter researchers from sharing their data openly, given the time and expertise involved, which would create an obstacle in advancing the goals of open science. However, in certain fields, like genomics, swift data sharing is expected (Rylance et al., 2016). The UK Concordat on Open Research Data provides that in order to encourage researchers to develop and share their data, those who create original data must be granted a reasonable right of exclusive first use for a suitable period, which is to be established through consultation and included in data management plans (Rylance et al., 2016). The right of creators of research data to reasonable first use is not a typical guiding principle for managing open access data. Open access encourages data sharing, but does not specify how data should be used prior to it being shared or the rights of the data creator (Fecher et al., 2015).

I suggest that the Draft Guidelines remove reference to the right to reasonable first use, and instead focus on ownership. In South Africa, the current position is that the data generator can acquire ownership of the data (Thaldar, 2024 forthcoming; Thaldar et al., 2022). Therefore, there is no need to deal with the right to reasonable first use in this context. Recent academic literature has established that in South African law, instances of data are susceptible of private ownership (Thaldar et al., 2022), and further, that research institutions are best positioned to claim ownership of these newly generated data instances (Thaldar, 2024 forthcoming). However, having ownership in research data instances does not mean that research institutions can do as they wish with the data. Research institutions will be subject to: (1) ethics oversight by a health research ethics committee; and (2) the provisions of POPIA (Thaldar, 2024 forthcoming).

It is important that the Draft Guidelines differentiate data ownership from copyright in datasets. While ownership of data is governed by property law—as found in South Africa’s common law—copyright in a dataset is governed by intellectual property law—specifically the Copyright Act 98 of 1978, 1978. Although these areas of law overlap, copyright in a dataset provides a layer of legal protection separate from ownership (Thaldar, 2024 forthcoming; Thaldar, et al., 2022; Swales, et al., 2023). In South Africa, the right of first use—or the exclusive right of use—features in copyright law. In terms of section 7(a) of the Copyright Amendment Bill (2018), where public funding is involved in research, the creator of the work may publicize it, even if an exclusive right of use exists. Therefore, it is clear that the focus of this principle lies in copyright and not ownership.

Being the data owner will assist in giving researchers the confidence that they have the right to openly share their data—thereby promoting open access and open science. As such, I suggest that this principle be replaced with the following: “Data generators, as owners of the data, should be encouraged to openly share their data”. This revised principle should explain: (1) the position on ownership of data in South African law; (2) the fact that ownership and intellectual property rights should not be confused; and (3) how data generators should promote open access and open science by sharing their data. Additionally, recognition should be given to indigenous people, in line with the CARE Principles. The Draft National Open Science Policy acknowledges that the CARE Principles deal with research that is not unethical or exploitative, and where the design of data ecosystems ensures that indigenous people benefit from such research (DSI, 2022). The Draft Guidelines contain a section on indigenous knowledge, but it does not deal with this in terms of data ownership and related ethical principles (NHREC, 2023). By overlooking data ownership in South Africa, I suggest that the Draft Guidelines are neglecting a vital aspect of open access data, which will only lead to further difficulties.

#### 3.5.3 Principle (3): for sound reasons, openness of research data may be restricted

The Draft Guidelines provide that the openness of research data may be limited if there are “sound reasons” for doing so (NHREC, 2023). However, it is unclear what constitutes a sound reason. This principle in the Draft Guidelines was adopted from the UK Concordat on Open Research Data (Rylance et al., 2016), which provides that, in certain circumstances, open access to research data may be restricted—for example, to protect privacy and confidentiality of participants, to avoid excessive costs, to uphold consent, to manage risks, to safeguard intellectual property rights, and to abide by other legal limitations (Rylance et al., 2016; Besançon et al., 2021). Moreover, the Organization for Economic Co-operation and Development (OECD) *Principles and Guidelines for Access to Research Data from Public Funding* (OECD, 2007) provide that access to, and use of, certain research data may be limited in some instances, such as national security, privacy and confidentiality, intellectual property rights, and legal processes (OECD, 2007). Governance arrangements, based on good practice and grounded in legal, regulatory, and ethical requirements, should be implemented to establish if and how data should be made openly available (Rylance et al., 2016). The UK Concordat on Open Research Data emphasizes that limitations on openness should not constitute a blanket ban, but should be determined on a case-by-case basis (Rylance et al., 2016). In terms of research publications, it has been suggested that, by default, data should be shared—with providing access to raw data as a prerequisite for manuscript submission. Where this is not possible, journal editors should request that the raw data is inspected by a reliable third party to verify the existence of the raw data and confirm the research results (Besançon et al., 2021).

In South Africa, publicly funded research is governed in part by the Intellectual Property Rights from Publicly Financed Research and Development Act 51 of 2008, 2008 (IPR Act). Section 2 of the IPR Act provides that intellectual property arising from publicly financed research must be protected, used, and commercialized in a way that benefits South Africa (Townsend et al., 2023). The Draft Guidelines refer to publicly funded research, stating that it is a public good and should be made openly available without imposing unwarranted or unjustifiable limitations (NHREC, 2023). The Draft National Open Science Policy applies to all publicly funded research, as well as data that is generated or acquired using public funds (DSI, 2022). In following the principle of “as open as possible, as closed as necessary,” certain research projects may entail licensing conditions—which will be determined on a case-by-case basis and by balancing open science and intellectual property licensing (DSI, 2022). Although public funders may have conditions regarding accessibility of the research in their contracts, these contracts do not override any statutory obligations to publicize research. Research funded by the private sector is often subject to contractual terms, but the Draft National Open Science Policy is to be applied in the best way possible, while respecting the private sector funding conditions (DSI, 2022). This is an example of an instance in which the openness of research data may be restricted. As such, I suggest that the Draft Guidelines elaborate on situations when the openness of research data may be restricted, what these sound reasons are, and how they will be implemented. Additionally, the guiding principles in the Draft Guidelines should promote open access wherever possible, rather than restrict it. In line with this, I suggest that the Draft Guidelines rephrase this principle to state that: “openness of research data should be promoted, wherever possible”. An explanation can be provided under this guiding principle with a caveat listing instances where openness may be restricted.

#### 3.5.4 Principle (4): a data management plan should be established at the start of the research process

A data management plan is a formal document that details how data will be handled throughout a research project. It addresses the data to be gathered during a research project, its management, analysis, and storage, as well as measures for sharing and preserving data once the research is complete (IBM, 2023; University of Pretoria, 2023). The Draft Guidelines recognize the importance of establishing a data management plan at the beginning of the research process. This is also provided for in the Draft National Open Science Policy, which requires data management plans for all publicly funded research in order to ensure long-term data curation and stewardship of open data (DSI, 2022). A way in which the Draft Guidelines can promote open science in its guiding principles for open access is to require that data management plans, where applicable, describe how data used in research will be made open—such as alignment with government standards and the principles of findability, accessibility, inter-operability, and re-usability (FAIR)—in line with, and as provided for in, the Draft National Open Science Policy (DSI, 2022).

Additionally, the POPIA Code of Conduct for Research contains the relevant information that researchers must include in their research protocol. A research protocol is defined as “documentation that outlines the plan of a research study” and is inclusive of a data management plan (ASSAf, 2023). These research protocols must encompass the data being collected and its purpose, safeguards, and data quality reviews (ASSAf, 2023). Given that the POPIA Code of Conduct for Research deals with health and genomics research, and given that it contains requirements for research protocols, I suggest that the Draft Guidelines make specific reference to the POPIA Code of Conduct for Research when dealing with data management plans. This will ensure that researchers are provided with further, and detailed, guidance that is in line with data protection laws in South Africa.

#### 3.5.5 Principle (5): use of secondary data should be governed by legal, ethical and regulatory frameworks that promote protection of personal information of donor/participants

The Draft Guidelines state that the use of secondary should be governed by legal, ethical, and regulatory frameworks that protect personal information, but fail to expand on what these frameworks are. For example, POPIA—as well as the POPIA Code of Conduct for Research—are specifically designed for this purpose, but are not mentioned in this section of the Draft Guidelines. Without concrete guidance and clarity, the guiding principles for open access data provided in the Draft Guidelines fall short.

Furthermore, it is not just the secondary use of data that is important. The initial processing of data must adhere to data protection laws. Section 13(1) of POPIA requires that personal information be collected for a “specific, explicitly defined and lawful purpose”. Section 15(1) of POPIA allows for the further processing of personal information, provided that it is compatible with the purpose for which it was originally collected. Therefore, if data was initially collected for research, any subsequent use of the data for research is allowed in terms of POPIA. Further, where personal information is used for *inter alia* research purposes, section 15(3) (*e*) of POPIA provides that further processing is compatible with the purpose of collection—as long as the information is only processed for research and is not published in an identifiable manner. If the processing involves special personal information—which is inclusive of genomic data—further processing is permitted, provided that it is for research and: (1) the research serves a public interest, which the processing is necessary for, or it would be unfeasible or involve an excessive effort to obtain consent; and (2) the responsible party can assure that the processing does not negatively and disproportionately impact the data subject’s privacy (section 27(1) (*d*) of POPIA). POPIA provides the primary protection for the use and secondary use of personal information, but the POPIA Code of Conduct for Research—which was developed to assist in ensuring legal certainty and compliance with the relevant provisions in POPIA (ASSAf, 2023) —offers additional guidance in this regard.

The POPIA Code of Conduct for Research is mentioned in the Draft Guidelines in terms of privacy and confidentiality of participants, and offers a means to ensure that researchers are compliant with POPIA (NHREC, 2023). But the POPIA Code of Conduct for Research is overlooked in terms of the secondary use of data. The POPIA Code of Conduct for Research deals with further processing (or secondary use). This occurs where the purpose for which the personal information is used changes, or the personal information is re-used for a different purpose (ASSAf, 2023). Where personal information collected for previous research is sought to be used for a different purpose, the researcher must provide certain information, including: (1) the circumstances under which the personal information was collected; (2) how assurances will be made that the personal information will only be used for research and will not be published in an identifiable manner; (3) how the notification requirement in section 18 of POPIA will be complied with; and (4) whether permission has been obtained from the responsible party who originally processed the personal information (ASSAf, 2023; Townsend et al., 2023).

The Draft Guidelines, while providing a principle regarding the protection of personal information, only consider secondary use of data (and not initial use) and fail to define the “legal, ethical and regulatory frameworks” that are applicable. This means that there is a lack of guidance regarding this important aspect of research, and which could lead to a contravention of the provisions in POPIA. To amend this, I suggest that the Draft Guidelines revise this guiding principle as follows: “the use and re-use of data should be governed by legal, ethical, and regulatory frameworks that promote the protection of personal information”. Additionally, I suggest that the Draft Guidelines: (1) provide for both the initial use, as well as the re-use, of data; and (2) make reference to POPIA and the POPIA Code of Conduct for Research. However, the Draft Guidelines should ensure that they state the law as it exists, rather than attempting to engage in an interpretive exercise.

#### 3.5.6 Principle (6): use of secondary data should include appropriate acknowledgement of the sources of their data and adhere to the terms of access and use

The final guiding principle for managing open access data in the Draft Guidelines provides that use of secondary data should acknowledge its sources and comply with the terms of access and use. This principle in the Draft Guidelines is taken from the UK Concordat on Open Research Data (Rylance et al., 2016). It is important for subsequent users of data to comply with any rules or restrictions placed on the data (Rylance et al., 2016). The UK Concordat on Open Research Data requires that researchers cite all data that they use in order to acknowledge the data source and creator (Rylance et al., 2016). Open access entails the sharing of data, which strengthens the usefulness and impact of data and increases accountability by allowing others to test analyses or utilize different methodologies to replicate findings (Devriendt et al., 2022). However, in order to ensure that open science is promoted, and researchers are incentivized to openly publish their data, original sources and creators should be acknowledged (Devriendt et al., 2022).

While the Draft Guidelines refer to the “use of secondary data,” most other policies and strategies in South Africa dealing with open science, open access, and open data refer to re-use. Although POPIA and the POPIA Code of Conduct for Research do not specifically require acknowledgement of the data source, it promotes transparency—a lawful ground for the processing of personal information in POPIA—and it is good practice to acknowledge sources.

The Draft National Open Science Policy, while not specifically referring to “secondary use,” does refer to “re-use” and permits data to be used and re-used freely without restriction, and without the need to acknowledge sources (DSI, 2022). On the other hand, both the Draft National Policy on Data and Cloud (Department of Communications and Digital Technologies, 2021) and the ICT Policy White Paper (Department of Telecommunications and Postal Services, 2016) are more restrictive in terms of the re-use of data. The Draft National Policy on Data and Cloud states that “data must be provided under terms that permit re-use and redistribution” (Department of Communications and Digital Technologies, 2021). Part of the definition of “open data” in the ICT Policy White Paper provides that datasets may be used and re-used, but “that users attribute the data and that they make their work available to be shared as well” (Department of Telecommunications and Postal Services, 2016). Moreover, one of the principles of the ICT Policy White Paper is that identified data “should be freely available for redistribution, use and re-use on conditions, including that the source of the data is identified, and that it is redistributed under the same terms and conditions” (Department of Telecommunications and Postal Services, 2016). However, the subsequent principle in the ICT Policy White Paper requires that data be legally open, meaning that it is in the public domain and can be used and re-used without restriction (Department of Telecommunications and Postal Services, 2016). Therefore, in terms of the re-use of data and acknowledgement of the original source, there seem to be conflicting views.

As good practice, I suggest that the Draft Guidelines amend this guiding principle to read as follows: “The re-use of data should include appropriate acknowledgement of the sources and adhere to the terms of access and use”. It is important for the Draft Guidelines to clarify what is meant by this guiding principle and what is required of researchers in this regard.

#### 3.5.7 Conclusion on the Draft Guidelines’ guiding principles for open access data

In determining guiding principles for open access data, the Draft Guidelines rely solely on the Draft National Policy on Data and Cloud to the exclusion of other relevant policies and documents. However, open data—as the Draft Guidelines define it—cannot be viewed in isolation, and regard must be had to the broader concept of open science. Open science and its related terms—such as open access and open data—feature in several government policies and strategies and offer potential pathways for the open sharing of data. Many of the existing policies and strategies do not provide concrete guidance on open science or open access, but rather call for the establishment of a policy or framework to govern the field (Department of Telecommunications and Postal Services, 2016; DSI, 2019; DST, 2019). However, there are those that are more detailed in offering objectives and principles for open science (including open access and open data). Below, I consider five main government documents—the ICT Policy White Paper, the Draft National Policy on Data and Cloud, the AOSP, the STI White Paper, and the Draft National Open Science Policy. I suggest that the Draft Guidelines be cognizant of these documents and incorporate certain principles, where relevant.

The ICT Policy White Paper aims to utilize Information and Communication Technologies (ICTs) to reduce poverty and inequality in South Africa (Department of Telecommunications and Postal Services, 2016). Part of the ICT Policy White Paper includes a focus on open government and open data. This entails that essential data is freely available, provided that privacy, confidentiality, and security are protected (Department of Telecommunications and Postal Services, 2016). The principles for open data include that: (1) making data open should be the norm, without violating an individual’s right to privacy and security; (2) data that is personal and confidential remains protected; (3) identified data should be freely available for redistribution, use, and re-use subject to certain conditions, including identification of the data source and redistribution under the same terms and conditions; (4) data must be available in the public domain without restriction and published in machine readable, non-proprietary formats; and (5) all data must be accessible and discoverable (Department of Telecommunications and Postal Services, 2016).

The Draft National Policy on Data and Cloud aims to promote the socio-economic value of data and create an enabling environment for the data ecosystem to flourish through *inter alia*: (1) the promotion of access to data and cloud services; (2) the establishment of measures for infrastructure protection; (3) the formation of governance mechanisms for data and cloud services; and (4) the provision of research and innovation (Department of Communications and Digital Technologies, 2021). The Draft National Policy on Data and Cloud recognizes that data should be equally available to all for its benefits to be realized, and that open data is vital in the data revolution (Department of Communications and Digital Technologies, 2021). As such, there is a need for an open data strategy in South Africa, informed by ‘Data for Good’ principles, to increase the accessibility of data (Department of Communications and Digital Technologies, 2021).

The AOSP recognizes that the shift to open science is necessary (AOSP, 2023). As such, the AOSP suggests the creation of an African Open Science Platform (the Platform) aimed at empowering African scientists with resources and principles for open science. This initiative is designed to foster scientific excellence and promote the practical application of scientific knowledge in various sectors. The AOSP envisions a platform that supports data-driven research focused on solutions, promoting collaboration between scientists and non-scientists within open networks. Through this collaborative approach, the AOSP aims to generate practical knowledge, enhance the credibility and relevance of science, and bolster its socio-political standing in Africa (AOSP, 2023). The AOSP aims to: (1) map the current data and science initiatives in Africa; (2) create a Pan-African open science community; and (3) develop frameworks to guide the Platform (AOSP, 2023). Given that science communities need to be large, diverse, and collaborative in order to succeed, the AOSP believes that the Platform should be Pan-African. Africa is diverse and this strength should be utilized in order to realize its potential. The AOSP suggests that an individual approach to science in Africa, especially where science communities are small and lack funding, would be a missed opportunity (AOSP, 2023).

Among the policy intents of the STI White Paper is ensuring that South Africa’s knowledge system is open, diverse, and responsive (DST, 2019). The STI White Paper recognizes the importance of transdisciplinary knowledge and the data-driven nature of research. Open science offers a solution for greater access to existing information and to benefit from collaborative and transdisciplinary approaches to knowledge development (DST, 2019). However, transitioning to open science requires suitable regulatory frameworks and the development of data skills (DST, 2019). The STI White Paper offers several measures that will be taken in adopting open science in South Africa. These include: (1) promoting open science incentives through education and researcher career development programs; (2) evaluating (and removing) barriers to open science and ensuring that legislation and practice support open science principles; (3) reviewing policies and institutions that govern access to research data and publications, and encouraging researchers to upload their data in public repositories and publish in open access journals; (4) identifying a license system for depositing, and using, open data; (5) respecting the data provider by determining who can use the data, and under what conditions; (6) a reconsideration of the IPR Act to ensure that it supports the findable, accessible, interoperable, and reusable (FAIR) guiding principles for the management and storage of data; (7) the development of a model for data storage and the cloud; and (8) the harmonization of data repositories (DST, 2019). Part of the intentions of the STI White Paper in terms of open science are to develop a framework containing guidelines and principles for open science in South Africa (DST, 2019). This resulted in the Draft National Open Science Policy.

Importantly, the Draft National Open Science Policy specifically provides guiding principles for open science in South Africa (DSI, 2022). The guiding principles for open science are based on the following core values: (1) quality and integrity through transparency, critique, and reproducibility; (2) equity, fairness, and collective benefit; and (3) diversity, collaboration, and inclusiveness (DSI, 2022). Additionally, there are guiding principles to assist in implementing open science in South Africa: (1) publicly funded data and results must be findable, accessible, inter-operable, and re-useable (FAIR); (2) cognisance of collective benefit, authority to control, responsibility, and ethics (CARE) principles, which deal with the ethical and non-exploitative framing of research; (3) the principles of transparency, responsibility, user community, and sustainability, and technology (TRUST) be taken into account when evaluating, developing, and maintaining the trustworthiness of data repositories; (4) a flexible approach to open science that is based on its context; (5) the open science model must be financially and operationally sustainable in the long-term; (6) the principles of “as open as possible, as closed as necessary” will be followed, which means that research outputs must be open and align with the objectives of the Draft National Open Science Policy, unless outweighed by other risks (DSI, 2022).

Based on the above, it is essentially only the Draft National Open Science Policy that explicitly provides guidelines for open science in South Africa. Although useful, it is clear that these guidelines are broad and are not tailored to the specific area of health research. Nevertheless, I suggest that the Draft Guidelines place greater reliance on the various government policies and strategies in existence as they are essential in the realization of open science in South Africa. The Draft Guidelines should be cautioned against adopting principles from other jurisdictions, as was done through reliance on the UK Concordat on Open Research Data (Rylance et al., 2016).

The AOSP highlights that, in adapting to open science, Africa should do so in its own way and based on its own priorities, rather than following other jurisdictions (AOSP, 2023). The AOSP recognizes that Africa should create its own open science platform, with the prospect of promoting science, society, and economic development (AOSP, 2023). A failure to do so will result in dependence on, and requiring skills from, other countries which will not serve to advance science and research (AOSP, 2023). As such, by using guiding principles from the UK Concordat on Open Research Data, the Draft Guidelines do little to serve and further the African agenda.

## 4 Suggestions for improving the Draft Guidelines

The guiding principles for managing open access data provided by the Draft Guidelines lack concrete guidance on a pathway for the use and sharing of open access data in health research in some respects. These guiding principles appear more as values that have little to do with promoting openness and access, and rather focus on the protection and limitation of such data. As such, there is certainly room for improvement, specifically in terms of the guiding principles for managing open access data. Below, I provide consolidated suggestions for improving the Draft Guidelines based on my analysis above.

### 4.1 Principle (1): strategies for long-term data curation are required

The suggestions for principle (1) are as follows: (1) provide a definition of “curation” in order to provide clarity to researchers; (2) remove reference to data curation in terms of POPIA as it does not appear in the Act; and (3) clarify how long-term value will be determined, or acknowledge that in the context of health research, it is likely that all data will be valuable in the long-term. The Draft Guidelines can explain that detailed long-term data curation may not be required for all research projects, and it depends on the research. Alternatively, this principle can be combined with principle (4) regarding data management plans below, in which case the Draft Guidelines can specify that long-term data curation be included as part of the data management plan or, where required and depending on certain factors like the nature of the research and the type of data collected, long-term data curation be detailed separately.

### 4.2 Principle (2): data generators, as owners of the data, should be encouraged to openly share their data

The suggestions for principle (2) are as follows: (1) remove reference to the right to reasonable first use, and instead focus on ownership; (2) explain the position on ownership of data in South African law; (3) differentiate data ownership from copyright in datasets; (4) promote the open sharing of data by data generators; and (5) recognition should be given to indigenous people and their data in terms of the CARE Principles.

### 4.3 Principle (3): openness of research data should be promoted, wherever possible

The suggestions for principle (3) are as follows: (1) elaborate on situations when the openness of research data may be restricted, what these sound reasons are, and how they will be implemented; and (2) provide an explanation under this guiding principle that contains a caveat listing instances where openness may be restricted.

### 4.4 Principle (4): a data management plan should be established at the start of the research process

The suggestions for principle (4) are as follows: (1) require that data management plans, where applicable, describe how data used in research will be made open; and (2) make specific reference to the POPIA Code of Conduct for Research, which contains requirements for research protocols.

### 4.5 Principle (5): the use and re-use of data should be governed by legal, ethical, and regulatory frameworks that promote the protection of personal information

The suggestions for principle (5) are as follows: (1) provide for both the initial use, as well as the re-use, of data; and (2) make reference to POPIA and the POPIA Code of Conduct for Research as the “legal, ethical and regulatory frameworks” that are applicable. The Draft Guidelines should be cautioned against interpreting the law, and should rather state the law as it exists.

### 4.6 Principle (6): the re-use of data should include appropriate acknowledgement of the sources and adhere to the terms of access and use

The suggestions for principle (6) are as follows: (1) remove reference to “secondary data” and replace it with “re-use”; and (2) clarify what is meant by this guiding principle and what is required of researchers in this regard.

In addition to the guiding principles for open access data, there are additional considerations that I suggest the Draft Guidelines take into account: (1) avoid placing sole reliance on the Draft National Policy on Data and Cloud and adopting principles from the UK Concordat on Open Research Data that may not apply in South Africa in their current form; (2) explicitly mention open science and expand on its importance in health research; (3) develop a comprehensive definition of “open data” that takes into account other definitions provided by the Draft National Open Science Policy and the ICT Policy White Paper; (4) provide other definitions relevant to open access and data in research, such as “open science” and “open access,” and differentiate between “open data,” “open access,” and “open access data”; (5) provide a potential pathway for open consent to further open science; (6) retain the previous provision in the 2015 DoH Ethics Guidelines regarding blanket consent to allow for the possibility of open consent; (7) refer to other South African government documents that deal with open science, open access, and open data to bolster the Draft Guidelines; and (8) include reference to South African legislation, where relevant. I also suggest that each of the guiding principles for managing open access data provided in the Draft Guidelines are accompanied by an explanation in order to expand on the principle and provide proper, and more detailed, guidance to researchers.

## 5 Conclusion

Health and genomics research in South Africa have a vital role to play in bettering the health of the population through an increased understanding of various diseases and the ability to develop more effective treatments and advance healthcare and technologies. However, its full potential cannot be realized if data and resources are not open and accessible to others. The Draft Guidelines serve to guide researchers in conducting health research in an ethical and responsible manner. Although the Draft Guidelines set the benchmark for health research in South Africa and are invaluable in certain respects, the inclusion of open access databases in the Draft Guidelines requires improvement. By only relying on one draft government policy—namely, the Draft National Policy on Data and Cloud—and overlooking other drafts that are relevant, such as the Draft National Open Science Policy, the Draft Guidelines cannot provide a comprehensive and context-specific pathway for open access data in research. Additionally, and from a policy perspective, the Draft Guidelines have an obligation to consider, and align with, principles of open science. By failing to expressly do so, the Draft Guidelines fall short in this regard.

While the Draft Guidelines and its inclusion of open access, especially in the context of health research, is a positive step towards open science and the transformation of the research landscape in South Africa, there is room for improvement. Specifically, the Draft Guidelines should: (1) specifically include reference to open science and its importance in South Africa; (2) add additional (and comprehensive) definitions for clarity, such as “open science” and “open access”; (3) consider the pathway for open access databases in South Africa by relying on an open consent model; and (4) have regard to the guiding principles for open access data and ensure that detailed guidance is provided to researchers, with reference being made to other relevant South African legislation and policy. The Draft Guidelines can also place reliance on existing policies and strategies that deal with open science and open access in order to align the Draft Guidelines with national imperatives. The implementation of these suggestions will serve to strengthen the Draft Guidelines and its position on open access databases.

## References

[B1] Abdool Karim Q. Baxter C. (2022). COVID-19: impact on the HIV and Tuberculosis response, service delivery, and research in South Africa. Curr. HIV/AIDS Rep. 19, 46–53. 10.1007/s11904-021-00588-5 35064888 PMC8783194

[B2] Abdool Karim S. S. Churchyard G. J. Abdool Karim Q. Lawn S. D. (2009). HIV infection and tuberculosis in South Africa: an urgent need to escalate the public health response. Lancet 374 (9693), 921–933. 10.1016/S0140-6736(09)60916-8 19709731 PMC2803032

[B3] Academy of Science of South Africa (ASSAf) Department of Science and Technology (DST). (2018). Human genetics and genomics in South Africa: ethical, legal and social Implications. 10.17159/assaf.2018/0033

[B4] Academy of Science of South Africa (ASSAf) (2019). African open science platform – Part 1: landscape study. Available at: https://aosp.org.za/wp-content/uploads/2022/05/aosplandscape-12aug2019_compressed.pdf.

[B5] Academy of Science of South Africa (ASSAf) (2023). POPIA Code of conduct for research. Available at: https://www.assaf.org.za/wp-content/uploads/2023/04/ASSAf-POPIA-Code-of-Conduct-for-Research.pdf.

[B6] African Open Science Platform (AOSP) (2023). The future of science and science for the future. Available at: https://aosp.org.za/wp-content/uploads/2023/05/AOSP-Strategy.pdf.

[B7] Angrist M. (2009). Eyes wide open: the Personal Genome Project, citizen science and veracity in informed consent. Per. Med. 6 (6), 691–699. 10.2217/pme.09.48 22328898 PMC3275804

[B8] Baca M. R. (2006). Barriers to innovation: intellectual property transaction costs in scientific collaboration. Duke L. Tech. Rev. 5, 1–14.

[B9] Begley C. Ellis L. (2012). Drug development: raise standards for preclinical cancer research. Nature 483, 531–533. 10.1038/483531a 22460880

[B10] Besançon L. Peiffer-Smadja N. Segalas C. Jiang H. Masuzzo P. Smout C. (2021). Open science saves lives: lessons from the COVID-19 pandemic. BMC Med. Res. Methodol. 21, 117–118. 10.1186/s12874-021-01304-y 34090351 PMC8179078

[B11] Buxton R. T. Nyboer E. A. Pigeon K. E. Raby G. D. Rytwinski T. Gallagher A. J. (2021). Avoiding wasted research resources in conservation science. Conservation Sci. Pract. 3, 1–11. 10.1111/csp2.329

[B12] Chakravorty N. Sharma C. S. Molla K. A. Pattanaik J. K. (2022). Open science: challenges, possible solutions and the way forward. Proc. Indian Natl. Sci. Acad. 88, 456–471. 10.1007/s43538-022-00104-2

[B13] Collins F. S. Varmus H. (2015). A new initiative on precision medicine. N. Engl. J. Med. 372 (9), 793–795. 10.1056/NEJMp1500523 25635347 PMC5101938

[B14] Copyright Act 98 of 1978, 1978 [South Africa].

[B15] Copyright Amendment Bill (2018) [South Africa].

[B16] Coresignal (2021). Data curation: benefits, goals, and best practices. Available at: https://coresignal.com/blog/data-curation/ (Accessed September 27, 2023).

[B17] Day S. Rennie S. Luo D. Tucker J. D. (2020). Open to the public: paywalls and the public rationale for open access medical research publishing. Res. Involv. Engagem. 6 (8), 8–7. 10.1186/s40900-020-0182-y 32161664 PMC7048123

[B89] Department of Science and Technology (DST) (2013). The bio-economy strategy. South Africa. Available at: https://www.gov.za/sites/default/files/gcis_document/201409/bioeconomy-strategya.pdf.

[B18] Department of Communications and Digital Technologies (2021). Draft national policy on data and cloud. GN 306 GG 44389. South Africa. Available at: https://www.gov.za/sites/default/files/gcis_document/202104/44389gon206.pdf.

[B19] Department of Health (DoH) (2015). Ethics in health research: principles, processes and Structures. South Africa. Available at: https://www.health.gov.za/wp-content/uploads/2022/05/NHREC-DoH-2015-Ethics-in-Health-Research-Guidelines-1.pdf.

[B20] Department of Science and Innovation (DSI) (2019). Synthesis report: South Africa Foresight exercise for science, technology and innovation 2030. South Africa. Available at: https://www.naci.org.za/wp-content/uploads/2020/07/South-African-Foresight-Exercise-For-Science-Technology-and-Innovation-2019.pdf.

[B21] Department of Science and Innovation (DSI). (2022). Draft national open science policy [South Africa].

[B22] Department of Science and Technology (DST) (2019). White paper on science, technology and innovation. South Africa. Available at: https://www.dst.gov.za/images/2019/White_paper_web_copyv1.pdf.

[B23] Department of Telecommunications and Postal Services (2016). National integrated ICT policy white paper. South Africa. Available at: https://www.gov.za/sites/default/files/gcis_document/201610/40325gon1212.pdf.

[B24] Devriendt T. Borry P. Shabani M. (2022). Credit and recognition for contributions to data-sharing platforms among cohort holders and platform developers in Europe: interview study. J. Med. Internet Res. 24 (1), e25983. 10.2196/25983 35023849 PMC8796038

[B25] European Commission (2023). What is open data. Available at: https://data.europa.eu/en/dataeuropa-academy/what-open-data (Accessed September 27, 2023).

[B26] Fecher B. Friesike S. (2014). “Open science: one term, five schools of thought,” in Opening science. Editor Bartling S. (Cham: Springer), 17–47.

[B27] Fecher B. Friesike S. Hebing M. (2015). What drives academic data sharing? PLoS ONE 10 (2), e0118053. 10.1371/journal.pone.0118053 25714752 PMC4340811

[B28] Friesike S. Widenmayer B. Gassmann O. Schildhauer T. (2015). Opening science: towards an agenda of open science in academia and industry. J. Technol. Transf. 40, 581–601. 10.1007/s10961-014-9375-6

[B29] Gewin V. (2016). Data sharing: an open mind on open data. Nature 529, 117–119. 10.1038/nj7584-117a 26744755

[B30] GIDA (2019). CARE principles for indigenous data governance. Available at: https://www.gida-global.org/care (Accessed January 24, 2024).

[B32] Gooden A. Thaldar D. (2023a). Toward an open access genomics database of South Africans: ethical considerations. Front. Genet. 14, 1166029–1166037. 10.3389/fgene.2023.1166029 37260770 PMC10228717

[B33] Gooden A. Thaldar D. (2023b). The time is ripe for a large-scale South African genome project. Johannesburg, South Africa: Mail and Guardian. Available at: https://mg.co.za/thoughtleader/opinion/2023-06-13-the-time-is-ripe-for-a-large-scale-south-african-genome-project/ (Accessed September 14, 2023).

[B34] Grant S. Wendt K. E. Leadbeater B. J. Supplee L. H. Mayo-Wilson E. Gardner F. (2022). Transparent, open, and reproducible prevention science. Prev. Sci. 23, 701–722. 10.1007/s11121-022-01336-w 35175501 PMC9283153

[B35] Hardwicke T. E. Mathur M. B. MacDonald K. Nilsonne G. Banks G. C. Kidwell M. C. (2018). Data availability, reusability, and analytic reproducibility: evaluating the impact of a mandatory open data policy at the journal *Cognition* . R. Soc. Open Sci. 5 (8), 180448–180518. 10.1098/rsos.180448 30225032 PMC6124055

[B36] Haven T. Gopalakrishna G. Tijdink J. van der Schot D. Bouter L. (2022). Promoting trust in research and researchers: how open science and research integrity are intertwined. BMC Res. Notes 15, 302–305. 10.1186/s13104-022-06169-y 36127719 PMC9487848

[B37] Heise C. Pearce J. M. (2020). From open access to open science: the path from scientific reality to open scientific communication. SAGE Open, 1–14. 10.1177/2158244020915900

[B38] Hetu M. Koutouki K. Joly Y. (2019). Genomics for all: international open science genomics projects and capacity building in the developing world. Front. Genet. 10, 95–99. 10.3389/fgene.2019.00095 30828348 PMC6384230

[B39] IBM (2023). What is a data management plan? Available at: https://www.ibm.com/topics/data-management-plan (Accessed September 27, 2023).

[B40] Intellectual Property Rights from Publicly Financed Research and Development Act 51 of 2008, 2008 [South Africa].

[B41] International Human Genome Sequencing Consortium Linton L. M. Birren B. Nusbaum C. Zody M. C. Baldwin J. (2001). Initial sequencing and analysis of the human genome. Nature 409, 860–921. 10.1038/35057062 11237011

[B42] Johnson L. F. May M. T. Dorrington R. E. Cornell M. Boulle A. Egger M. (2017). Estimating the impact of antiretroviral treatment on adult mortality trends in South Africa: a mathematical modelling study. PLoS Med. 14 (12), e1002468. 10.1371/journal.pmed.1002468 29232366 PMC5726614

[B43] Krishna V. V. (2020). Open science and its enemies: challenges for a sustainable science–society social contract. J. Open Innov. Technol. Mark. Complex. 6 (3), 61–15. 10.3390/joitmc6030061

[B44] Kubjane M. Osman M. Boulle A. Johnson L. F. (2022). The impact of HIV and tuberculosis interventions on South African adult tuberculosis trends, 1990-2019: a mathematical modeling analysis. Int. J. Infect. Dis. 122, 811–819. 10.1016/j.ijid.2022.07.047 35872098 PMC9439958

[B45] Lee D. J. Stvilia B. (2017). Practices of research data curation in institutional repositories: a qualitative view from repository staff. PLoS One 12 (3), e0173987. 10.1371/journal.pone.0173987 28301533 PMC5354423

[B46] Levy E. Marden E. Warren B. Hartell D. Filaté I. (2010). Patent pools and genomics: navigating a course to open science? BU. J. Sci. Tech. L. 16, 75–101.

[B47] Lunshof J. E. Bobe J. Aach J. Angrist M. Thakuria J. V. Vorhaus D. B. (2010). Personal genomes in progress: from the human genome project to the personal genome project. Dialogues Clin. Neurosci. 12 (1), 47–60. 10.31887/DCNS.2010.12.1/jlunshof 20373666 PMC3181947

[B48] Lunshof J. E. Chadwick R. Vorhaus D. B. Church G. M. (2008). From genetic privacy to open consent. Nat. Rev. Genet. 9 (5), 406–411. 10.1038/nrg2360 18379574

[B49] Maurer S. M. (2003). “New institutions for doing science: from databases to open source biology,”. 19 November 2003 (University of Maastricht) in European policy for intellectual property conference on copyright and database protection, patents and research tools, and other challenges to the intellectual property system (Netherlands). Available at: http://www.merit.unimaas.nl/epip/papers/maurer_paper.pdf.

[B50] Mayosi B. M. Lawn J. E. van Niekerk A. Bradshaw D. Abdool Karim S. S. Coovadia H. M. (2012). Health in South Africa: changes and challenges since 2009. Lancet 380 (9858), 2029–2043. 10.1016/S0140-6736(12)61814-5 23201214

[B51] McKiernan E. C. Bourne P. E. Brown C. T. Buck S. Kenall A. Lin J. (2016). How open science helps researchers succeed. Elife 5, e16800–e16819. 10.7554/eLife.16800 27387362 PMC4973366

[B52] Miller O. (2023). 5 steps to creating a data curation plan. *Medium* . Available at: https://medium.com/@oliviamiller048/5-steps-to-creating-a-data-curation-plan-ffe784789c4a (Accessed September 27, 2023).

[B53] National Health Research Ethics Council (2023). South African ethics in health research guidelines: principles, processes and Structures. Available at: https://www.samrc.ac.za/research/rio-hrec-guideline-documents.

[B54] Nielsen M. (2011). Reinventing discovery: the new era of networked science. New Jersey: Princeton University Press.

[B55] NIH (2023). Data curation. Available at: https://www.nnlm.gov/guides/data-glossary/data-curation (Accessed September 27, 2023).

[B56] Nosek B. A. Alter G. Banks G. C. Borsboom D. Bowman S. D. Breckler S. J. (2015). SCIENTIFIC STANDARDS. Promoting an open research culture. Science 348, 1422–1425. 10.1126/science.aab2374 26113702 PMC4550299

[B57] Open Data Charter (2023). International open data charter. Available at: https://opendatacharter.net/principles/ (Accessed September 27, 2023).

[B58] Open Data Handbook (2023). What is open data. Available at: https://opendatahandbook.org/guide/en/what-is-open-data/ (Accessed September 27, 2023).

[B59] Open Knowledge Foundation. (2023). Open data. Available at: https://okfn.de/en/themen/open_data/#:∼:text=Open%20data%20is%20data%20that,not%20apply%20to%20personal%20data. (Accessed September 27, 2023).

[B60] Organization for Economic Co-operation and Development (OECD). (2007). Principles and guidelines for access to research data from public funding.

[B61] Podsakoff P. M. MacKenzie S. B. Podsakoff N. P. (2016). Recommendations for creating better concept definitions in the organizational, behavioral, and social sciences. Organ. Res. Methods 19 (2), 159–203. 10.1177/1094428115624965

[B62] Prainsack B. Buyx A. (2013). A solidarity-based approach to the governance of research biobanks. Med. Law Rev. 21 (1), 71–91. 10.1093/medlaw/fws040 23325780

[B63] Protection of Personal Information Act 4 of 2013, 2013 [South Africa].

[B64] Ramachandran R. Bugbee K. Murphy K. (2021). From open data to open science. Earth Space Sci. 8, 1–17. 10.1029/2020EA001562

[B65] Regulations Relating to Research with Human Participants GN R719 of GG 38000, 2014 [South Africa].

[B66] Rhoten D. Powell W. W. (2007). The frontiers of intellectual property: expanded protection versus new models of open science. Annu. Rev. Law Soc. Sci. 3, 345–373. 10.1146/annurev.lawsocsci.3.081806.112900

[B67] Rylance R. Wingham D. Wright N. Bruce R. Hammonds W. Arrowsmith J. (2016). Concordat on open research data. Available at: https://www.ukri.org/wp-content/uploads/2020/10/UKRI-020920-ConcordatonOpenResearchData.pdf.

[B68] Scaria A. G. Rangarajan R. (2016). Fine-tuning the IP approaches for fostering open science: some insights from India. WIPO J. 8 (1), 109–122. 10.2139/ssrn.2844625

[B69] Scott A. (2017). What is open data and why should we care. London, United Kingdom: Open Data Institute. Available at: https://www.theodi.org/article/what-is-open-data-and-why-should-we-care/ (Accessed September 27, 2023).

[B70] Smith E. Haustein S. Mongeon P. Shu F. Ridde V. Larivière V. (2017). Knowledge sharing in global health research: the impact, uptake and cost of open access to scholarly literature. Health Res. Policy Syst. 15 (1), 73–10. 10.1186/s12961-017-0235-3 28851401 PMC5576373

[B71] Spellman B. Gilbert E. A. Corker K. S. (2017). Open science: what, why, and how. PsyArXiv, 1–84. 10.31234/osf.io/ak6jr

[B72] Staunton C. Barragán C. A. Canali S. Ho C. Leonelli S. Mayernik M. (2021). Open science, data sharing and solidarity: who benefits? Hist. Philos. Life Sci. 43 (4), 115–118. 10.1007/s40656-021-00468-6 34762203 PMC8582236

[B73] Strydom A. Mellet J. Van Rensburg J. Viljoen I. Athanasiadis A. Pepper M. S. (2022). Open access and its potential impact on public health – a South African perspective. Front. Res. Metr. Anal. 7, 975109–975118. 10.3389/frma.2022.975109 36531754 PMC9755351

[B74] Swales L. Botes M. Donnelly D. Thaldar D. (2023). Towards a data transfer agreement for the South African research community: the empowerment approach. S. Afr. J. Bioeth. Law 16 (1), 13–18. 10.7196/SAJBL.2023.v16i1.827 37377981 PMC10299799

[B75] Thaldar D. (forthcoming) (2024). The wisdom of claiming ownership of human genomic data: a cautionary tale for research institutions. 10.1111/dewb.12443 PMC11289161 38298031

[B76] Thaldar D. Gooden A. Donnelly D. (2023a). Toward an open access genomics database of South Africans: legal considerations. S. Afr. J. Sci. 119 (7/8), 1–4. 10.17159/sajs.2023/15069 PMC10228717 37260770

[B77] Thaldar D. Gooden A. Steytler M. (2023b). Open science and human genetic data: recommendations on South Africa's draft national open science policy. Front. Genet. 14, 1248747. 10.3389/fgene.2023.1248747 37849503 PMC10577200

[B78] Thaldar D. Townsend B. (2020). Genomic research and privacy: a response to Staunton et al. S. Afr. Med. J. 110 (3), 172–174. 10.7196/SAMJ.2020.v110i3.14431 32657691

[B79] Thaldar D. W. Townsend B. A. Donnelly D. L. Botes M. Gooden A. van Harmelen J. (2022). The multidimensional legal nature of personal genomic sequence data: a South African perspective. Front. Genet. 13, 997595–997611. 10.3389/fgene.2022.997595 36437942 PMC9681828

[B80] Townsend B. Gooden A. Botes M. Thaldar D. (2023). Repurposing research data for commercial use: POPIA, a foil or a facilitator? S. Afr. J. Sci. 119 (7/8), 1–5. 10.17159/sajs.2023/15075 PMC12520580 41098943

[B81] UCL (2024). 8 pillars of open science. Available at: https://www.ucl.ac.uk/library/open-science-research-support/open-science/8-pillars-open-science#:∼:text=The%208%20pillars%20of%20Open,Rewards%20and%20Initiatives%2C%20and%20EOSC (Accessed January 24, 2024).

[B82] UCLA (2023). Digital humanities. Available at: https://guides.library.ucla.edu/c.php?g=180354&p=9650260 (Accessed September 27, 2023).

[B83] University of Pretoria (2023). Research data management (RDM): data management plans. Available at: https://library.up.ac.za/c.php?g=356288&p=2412447 (Accessed September 26, 2023).

[B84] Venter J. C. Adams M. D. Myers E. W. Li P. W. Mural R. J. Sutton G. G. (2001). The sequence of the human genome. Science 291 (5507), 1304–1351. 10.1126/science.1058040 11181995

[B85] Vicente-Saez R. Martinez-Fuentes C. (2018). Open Science now: a systematic literature review for an integrated definition. J. Bus. Res. 88, 428–436. 10.1016/j.jbusres.2017.12.043

[B86] Wang S. Jiang X. Singh S. Marmor R. Bonomi L. Fox D. (2017). Genome privacy: challenges, technical approaches to mitigate risk, and ethical considerations in the United States. Ann. N. Y. Acad. Sci. 1387 (1), 73–83. 10.1111/nyas.13259 27681358 PMC5266631

[B87] Whitfield G. (2012). The importance of proper definition. Available at: https://piadvice.wordpress.com/2012/06/13/the-importance-of-proper-definition/ (Accessed September 23, 2023).

[B88] Wilkinson M. Dumontier M. Aalbersberg I. Appleton G. Axton M. Baak A. (2016). The FAIR Guiding Principles for scientific data management and stewardship. Sci. Data 3, 160018–160019. 10.1038/sdata.2016.18 26978244 PMC4792175

